# Scribble deficiency mediates colon inflammation by inhibiting autophagy-dependent oxidative stress elimination

**DOI:** 10.1038/s41598-023-45176-2

**Published:** 2023-10-26

**Authors:** Xia Sun, Liying Lu, Kai Wang, Lele Song, Jiazheng Jiao, Yanjun Wu, Xinyu Wang, Yanan Song, Lixing Zhan

**Affiliations:** 1 CAS Key Laboratory of Nutrition, Metabolism and Food Safety, Shanghai Institute of Nutrition and Health, Shanghai Institutes for Biological Sciences, University of Chinese Academy of Sciences, Chinese Academy of Sciences, Shanghai, China; 2 Key Laboratory of Integrated Oncology and Intelligent Medicine of Zhejiang Province, Department of Hepatobiliary and Pancreatic Surgery, Affiliated Hangzhou First People’s Hospital, Zhejiang University School of Medicine, Hangzhou, 310006 China

**Keywords:** Cell biology, Molecular biology, Stem cells, Gastroenterology

## Abstract

Scribble is a master scaffold protein in apical-basal polarity. Current knowledge about the biological function of Scribble in colonic epithelial plasticity/regeneration during intestinal inflammation is limited. Here, we showed that the level of Scribble is decreased in inflammatory bowel disease (IBD) patients and mice with DSS-induced colitis. Scrib^ΔIEC^ mice develops severe acute colitis with disrupted epithelial barrier integrity and impaired crypt stem cell’s function. Mechanistically, Scribble suppressed the process of autophagy by modulating the stability of caspase-dependent degradation of Atg16L1 by directly interacting with Atg16L1 in a LRR domain-dependent manner in IECs and led to an accumulation of ROS both in intestinal stem cells and epithelial cells. In addition, further study indicates that dietary sphingomyelin alleviates DSS-induced colitis by increase the expression of Scribble, which suggests that Scribble may be the critical marker of IBD. Our study shows that Scribble deficiency is associated with the dysregulated autophagy and impaired maintenance of colonic stemness, and it may be a target for diagnosis and treatment of IBD.

## Introduction

Inflammatory bowel disease (IBD) is a common chronic intestinal disease, which include Crohn's disease (CD) and ulcerative colitis (UC). IBD is one of the most common gastrointestinal diseases in the world^1^. The incidence of IBD is increasing, and the number of IBD patients in China is projected to reach to 1.5 million by 2025^2^. Loss of gut barrier function, increased immune cell recruitment, and excessive immune response to the host gut microbiota are thought to contribute to the development of IBD^3,4^, but the exact etiology and pathogenesis of IBD are still poorly understood. Therefore, it is necessary to study the pathogenesis of IBD.

Intestinal barrier integrity plays an important role in resisting the invasion of pathogenic microorganisms. Loss of intestinal epithelial barrier function is a common feature of IBD, leading to inflammatory responses and barrier disruption^5^. The junction between intestinal epithelial cells (IECs) is the most critical feature of the intestinal barrier^6^, and incorporates tight junctions, adhesion junctions, and desmosomes^7^. IECs play an important role in maintaining the homeostasis and function of the intestinal environment^6^. The maintenance and differentiation of the intestinal stem cells (ISCs) are critical for IEC homeostasis and IBD repair. ISCs are located at the bottom of the crypt, where these cell proliferate and differentiate to generate all types of IECs^8^. Inner lineage tracing reveals that ISCs specifically express the leucine-rich repeat (LRR) -containing G protein-coupled receptor 5 (Lgr5)^9,10^. Wingless-intergrin (Wnt)/β-catenin signaling is essential for the maintenance of stem cell function, and contributes to controlling the expansion and differentiation of ISCs in response to tissue injury and repair in IBD^11,12^.

Autophagy is a highly conserved cellular process leading to the degradation of dysfunctional or damaged proteins and organelles^13^. At present, most of the evidence for a link between autophagy and IBD comes from studies of *Autophagy gene 16L1* (ATG16L1) and its protein product^14^. Earlier studies revealed that human cells harboring the *ATG16L1*^*T300A*^ mutation exhibit impaired clearance of bacteria and increased production of proinflammatory cytokines^15^, and Paneth cells from CD patients have been shown to be homozygous for *ATG16L1*^*T300A*14^ Mice engineered to express the human *ATG16L1*^*T300A*^ variant or to selectively lack expression of the mouse homolog (*Atg16L1*) in IECs (*Atg16L1*^*ΔIEC*^) exhibit abnormalities in the structure and function of Paneth cells, leading to decreased expression of antimicrobial peptide^14^. *ATG16L1*^*T300A*^ mice show impaired autophagy, a phenotype that reflects the fact that ATG16L1^T300A^ is more sensitive to caspase 3-mediated cleavage and degradation than is the wild-type protein^16,17^. *Atg16L1*^*ΔIEC*^ mice exhibit excessive necroptosis and tumor necrosis factor alpha (TNF-α) -mediated apoptosis in IECs, leading to exacerbation of colitis^18,19^. In summary, these previous studies show that Atg16L1 plays a critical role in controlling intestinal epithelial homeostasis and inflammatory immune responses Other Except autophagy-related genes (such as *Unc-51-like kinase 1* (*ULK1*), *Leucine-rich repeat kinase 2* (*LRRK2*), and *Tyrosine-protein phosphatase non-receptor type 2* (*PTPN2*) also have been shown to contribute to IBD, but, in contrast to the case with *ATG16L1*, the precise mechanism for the role of these other genes remain unknown^14^.

The Scribble scaffold protein was originally identified based on the molecule’s role in epithelial cell polarity and epithelial integrity in *Drosophila*. Scribble is a conserved polarity protein belonging to the LAP (LRR and PDZ) family, the protein includes 16 leucine-rich repeat (LRR) domains and 4 PSD-95, Discs-large, ZO-1 (PDZ) domains^20–22^. Scribble acts as an adaptor protein by facilitating key molecular interactions at distinct subcellular locations. The LRR protein SHOC2/SUR8 has been shown to interact with the LRR domains of Erbin and Scribble, forming an inhibitory complex for extracellular signal-regulated kinase (ERK) signaling ^23,24^. In some biological contexts, the region of Scribble containing the LRRs region is sufficient to rescue Scribble function^25–27^. Scribble PDZ domains also serve as key integration sites for molecular interactions with other proteins, organizing a vast range of cellular functions^28,29^, and Scribble PDZ domains show high affinity with numerous proteins such as β-PIX and GIT1^30^. Our previous study showed that Scribble cooperates with the Myc oncoprotein in a βPIX/ARGHEF7- and RAC-JNK-dependent manner^31^. Together, research results have revealed that Scribble could regulates the RAS-MAPK, TNF-JNK, PI3K-Akt, Hippo, and Wnt signaling pathways^32^. However, the potential roles of Scribble in the maintenance and regeneration of colonic barrier function, especially in the context of autophagy and the pathogenesis of IBD, have not yet (to our knowledge) been studied. In the present work, we demonstrated that Scribble inhibits colonic inflammation by ensuring that the process of autophagy proceeds, suggesting that Scribble serves as a key marker of the intestinal barrier.

## Results

### Scribble is decreased in IBD patients and mice with DSS-induced colitis

**Figure 1:**
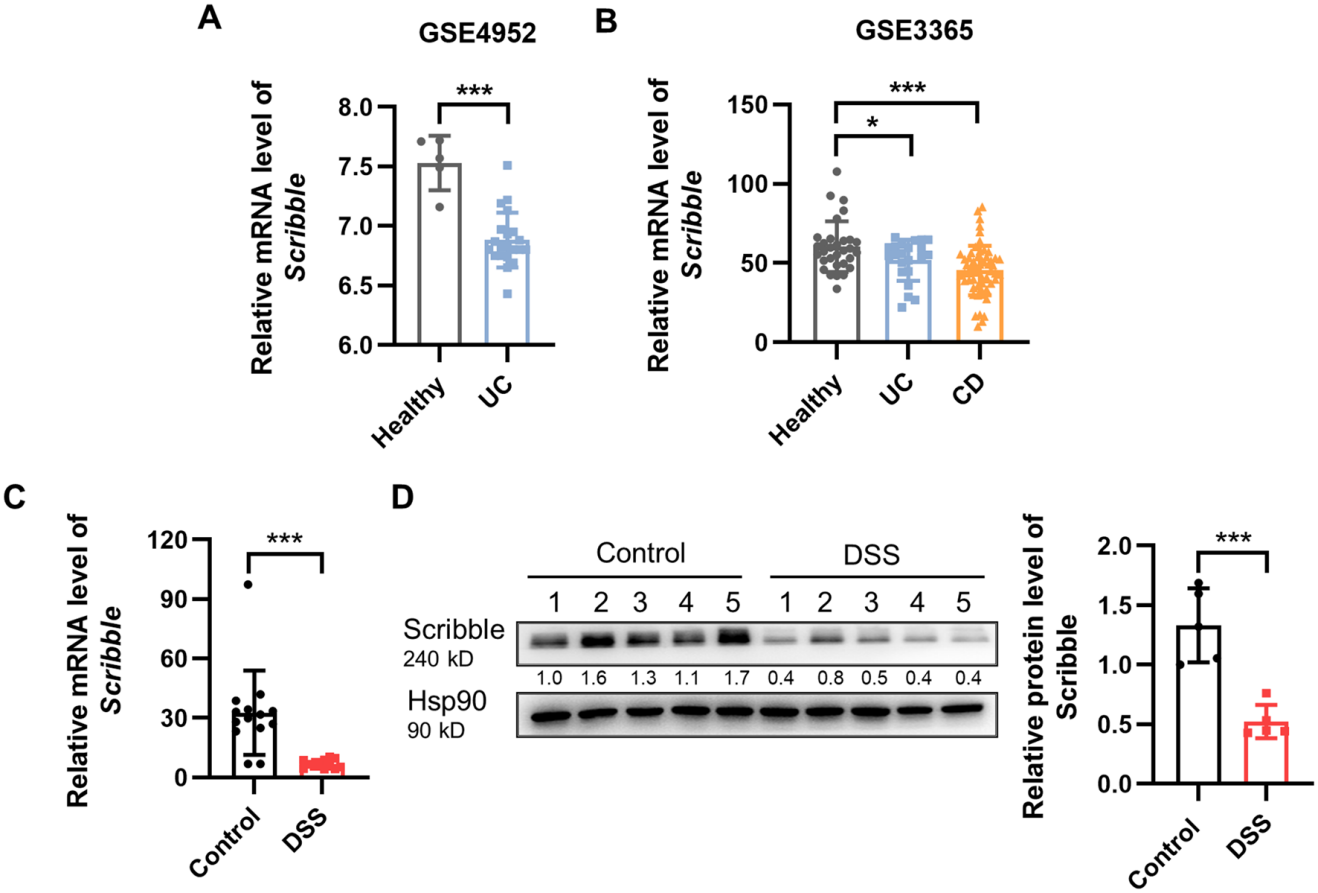
*Scribble* transcript and protein levels are decreased in IBD patients and in mice with DSS-induced colitis. (**A,B**) The relative expression of *Scribble* in specimens from healthy controls and from patients with IBD (**A** from GEO dataset GSE9452, **B** from GEO dataset GSE3365). (**C,D**) The relative mRNA (**C**) and protein (**D**) expression of *Scribble* in colonic tissues of mice maintained on drinking water neat (Control) or supplemented with 3% DSS (DSS). The data are presented as mean ± SEM. Statistical analyses were conducted using two-tailed unpaired Student’s t-tests, *p < 0.05, **p < 0.01, ***p < 0.001.

To determine whether Scribble is involved in the pathogenesis of IBD, we measured expression of this gene in specimens from patients with IBD and from healthy controls; this analysis employed datasets from public databases of NCBI GSE9452 and GSE3365. *Scribble* transcript levels were significantly lower in patients with CD and UC compared to control individuals (Fig. 1A,B). To further define the role of Scribble in the development of colitis, we employed an animal model of acute colitis induced by treatment of C57BL/6 mice with 3% dextran sodium sulfate (DSS). Specifically, the mice were divided into two groups and provided with drinking water, either neat (Control) or containing 3% DSS (DSS) (Supplementary Fig. 1A). After 7 days, mice were euthanized and gastrointestinal tissues were evaluated histopathologically. Mice of the + DSS group mice showed severe ulceration, destruction of crypt structure, and mucosal and submucosal lesions (Supplementary Fig. 1^1^). The protein levels of phosphorylated-JNK (p-JNK), phosphorylated-P65 (p-P65), phosphorylated-STAT3(p-STAT3) and the levels of transcripts encoding inflammatory cytokines were significantly elevated in colonic tissues from the + DSS treated animals compared to the -DSS mice (Supplementary Fig. 1C,D), confirming the successful induction of colitis in this model. Notably, the expression of *Scribble* (both at the mRNA and proteins levels) was significantly lower in the colonic tissues of mice with DSS-induced colitis compared to those from control animals (Fig. 1C,D), a result consistent with that obtained from the analysis of data from clinical samples. Thus, our data indicated that Scribble may play a role in the pathogenesis of IBD.

### Loss of Scribble in IECs increases the susceptibility of DSS-induced colitis

To clarify the potential role of Scribble in the pathogenesis of colitis, we generated an IEC-conditional *Scribble* knockout mouse line by mating *Villin*-*cre* mice (which express the Cre recombinase under control of the gut epithelial cell-specific *Villin* promoter) with *Scrib*^flox/flox^ mice (*Scrib*^f/f^; in which exons 2–8 of the *Scribble* gene are flanked by *loxP* recombination sites. The resulting animals, here referred to as *Scrib*^ΔIEC^ mice, demonstrate selective excision and inactivation of the *Scribble* gene in the IECs. (Supplementary Fig. 2A,B). As expected, the progeny of this cross exhibited *Scrib*^ΔIEC^ and *Scrib*^f/f^ genotypes in accordance with a Mendelian ratio; the littermate *Scrib*^f/f^ mice were used as controls. Western blot and qRT-PCR results confirmed efficient Scribble deletion in the intestine of the *Scrib*^ΔIEC^ mice (Supplementary Fig. 2C,D). Under otherwise identical growth conditions, the 2-month-old *Scrib*^ΔIEC^ mice displayed no significant differences compared to control mice. However, 8-month-old *Scrib*^ΔIEC^ mice exhibited lower body weight and shorter colon length than littermate control (Supplementary Fig. 3A,B). Furthermore, histopathology using hematoxylin and eosin staining at 8 months confirmed that crypt foci were sparser in *Scrib*^ΔIEC^ mice than in control animals (Supplementary Fig. 3C), although the *Scrib*^ΔIEC^ mice had not developed spontaneous enteritis.

**Figure 2:**
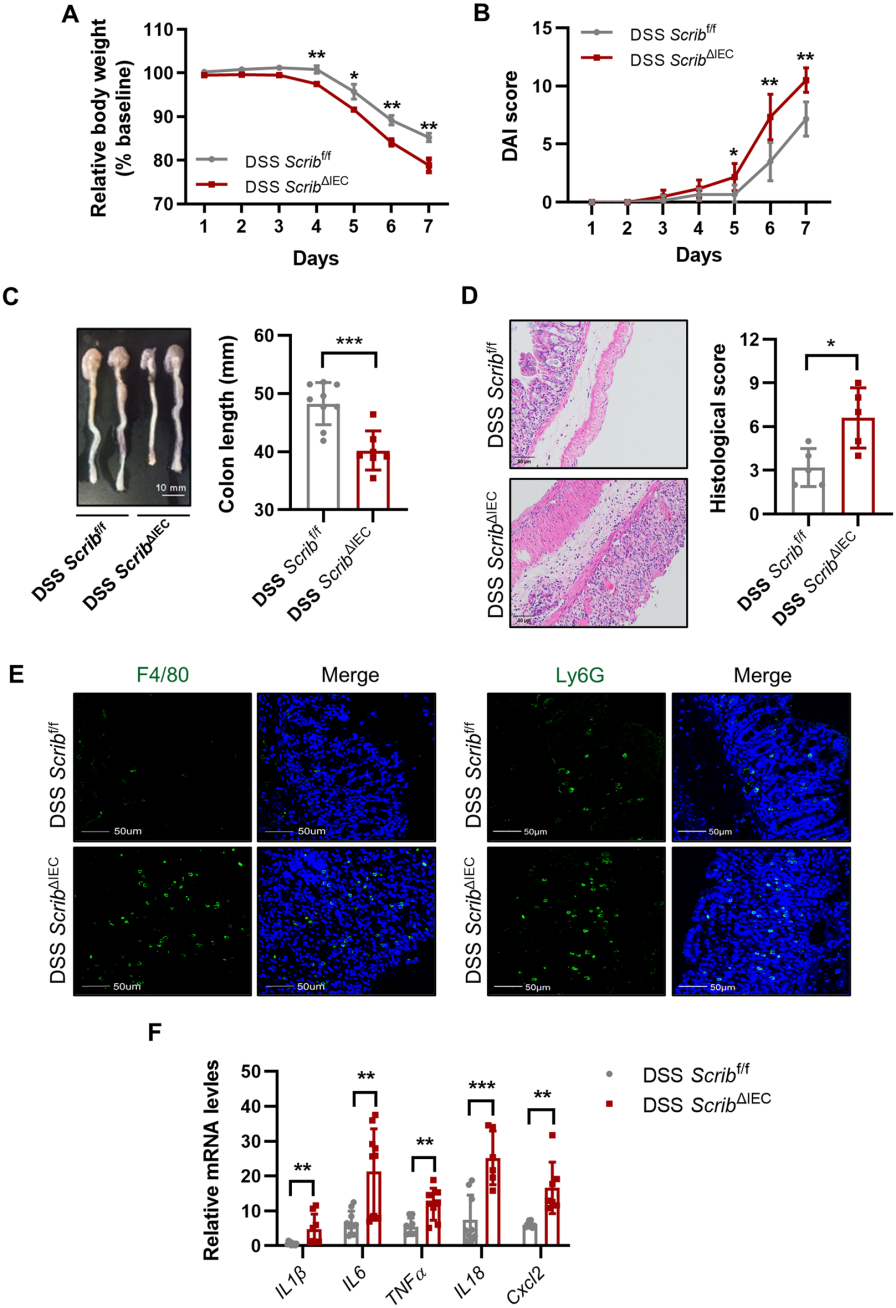
Loss of *Scribble* in IECs increases the susceptibility of DSS-induced colitis. *Scrib*^f/f^ and *Scrib*^ΔIEC^ mice (8 weeks old) were provided with free access to drinking water containing 2.5% DSS; after 7 days, the animals were euthanized. (**A**) Relative body weights (normalized to baseline). (**B**) DAI scores. (**C**) Representative images of colons from animals of the two groups and plot of colon length data (scale bar: 10 mm). (**D**) Representative H&E images of colon tissue and plot of colon histological scores (scale bar: 50 μm). (**E**) Representative images of immunofluorescence (IF) staining of macrophage marker F4/80 and the neutrophil marker Ly-6G (scale bar: 50 μm). IF staining is visualized as green punctae (F4/80^+^ or Ly6G^+^), alone and merged with DAPI staining for DNA (blue). (**F**) Relative expression in the colon mucosa of genes encoding inflammatory cytokines, as assessed by qRT-PCR. Expression levels were normalized to that of the housekeeping gene *β-actin.* The data are presented as mean ± SEM; comparisons were conducted by two-tailed unpaired Student’s t-test. *p < 0.05, **p < 0.01, ***p < 0.001.

Next, we induced colitis in 8-week-old *Scrib*^ΔIEC^ mice and *Scrib*^f/f^ mice by providing 7 days of free access to drinking water containing 2.5% DSS. Following induction with DSS, *Scrib*^ΔIEC^ mice exhibited significantly lower body weights, elevated disease activity index (DAI) scores, and shorter colon lengths compared to *Scrib*^f/f^ mice (Fig. 2A-C). Additionally, HE staining showed that DSS-exposed *Scrib*^ΔIEC^ mice exhibited more severe ulceration, mucosal epithelial denudation, crypt loss, and immune cell infiltration in the colon (Fig. 2D). Furthermore, the colons of *Scrib*^ΔIEC^ mice with DSS-induced colitis had significantly increased aggregation of macrophages (F4/80^+^ cells) and neutrophils (Ly-6G^+^ cells) in compared to those of *Scrib*^f/f^ mice (Fig. 2E). Consistent with these inflammatory changes, qualitative reverse transcriptase-polymerase chain reaction (qRT-PCR) analysis showed that transcripts encoding the pro-inflammatory cytokines IL-1β, IL6, TNFα, and IL-18, as well as that encoding the neutrophil-associated chemokine Cxcl2, accumulated to significantly higher levels in the colonic tissues of *Scrib*^ΔIEC^ mice than in those of controls (Fig. 2F). Together, these results highlighted our inference that Scribble plays a critical role in maintaining colonic homeostasis, and demonstrated that *Scribble* deletion in IECs potentiates the progression of DSS-induced colitis.

### Loss of *Scribble* in IECs impaires epithelial barrier integrity in DSS-induced colitis

**Figure 3:**
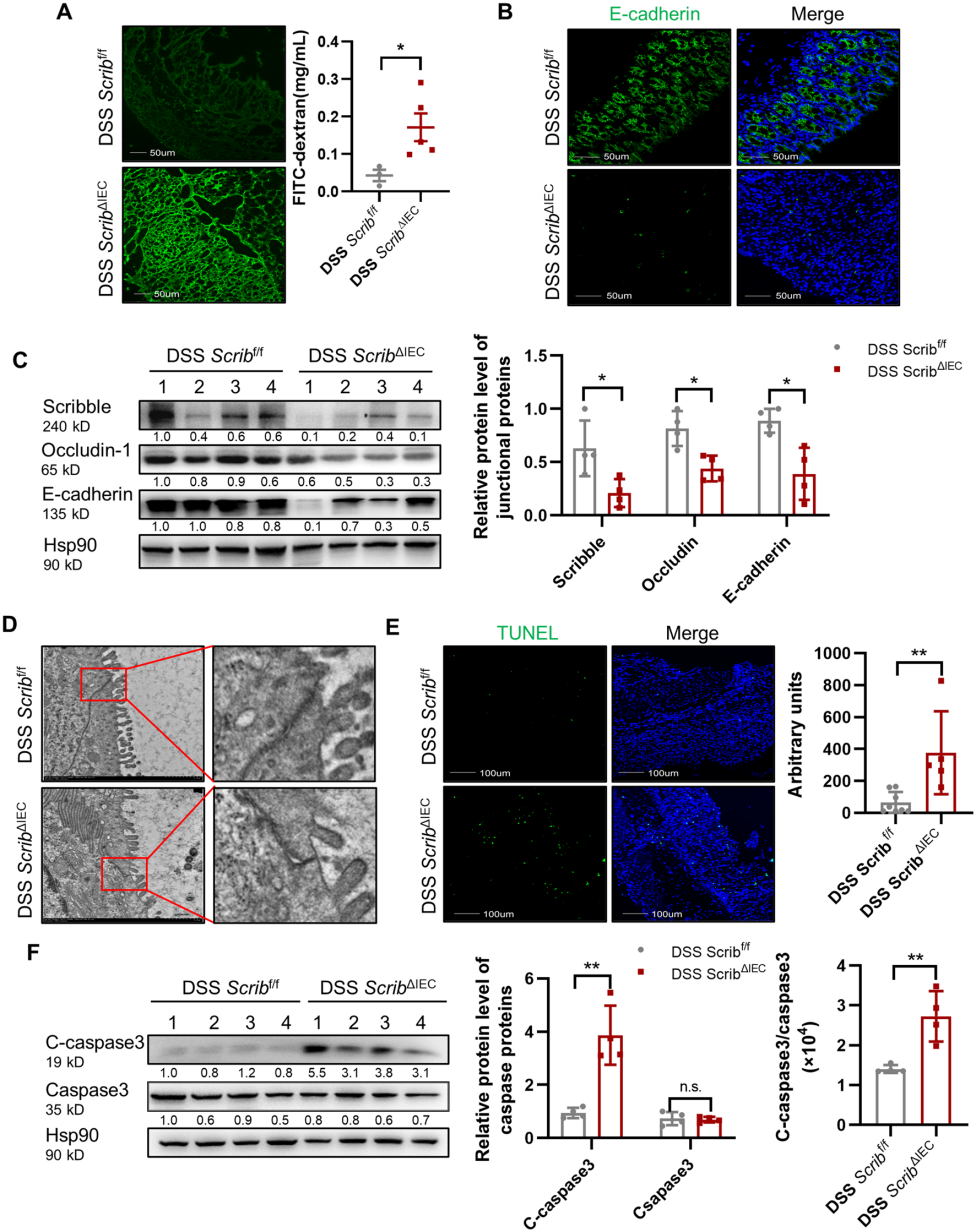
Loss of *Scribble* in IECs impairs epithelial barrier integrity in DSS-induced colitis. *Scrib*^f/f^ and *Scrib*^ΔIEC^ mice (8 weeks old) were provided with free access to drinking water containing 2.5% DSS; after 7 days, the animals were euthanized. (**A**) Representative images of fluorescence from FITC-dextran accumulation in the surface of the colon mucosa and graph of data for the level of FITC-dextran in serum. (**B**) Immunofluorescence (IF) staining for the epithelial marker E-cadherin (green) in the surface of the colon mucosa, both alone and merged with DAPI staining for DNA (blue). (**C**) Left: Expression of tight junction proteins (E-cadherin and occludin-1) and Scribble in colon tissues, as assessed by western blot using antibodies against the respective proteins. The levels of Hsp90, a housekeeping protein, were measured as a loading control. Right: Graph of data for relative protein expression, as obtained by measuring (using ImageJ) the band intensity in the western blot; values were normalized to those for the Hsp90 loading control in the respective lane. (**D**) Representative transmission electron microscope (TEM) images of tight junctions in colon epithelial cells. Images on the right are enlarged from the boxes indicated in the images on the left. (**E**) TUNEL assay for colon tissue of mice. Left: Representative images of TUNEL staining (green) in the surface of the colon mucosa, both alone and merged with DAPI staining for DNA (blue). Right: Graph of data for TUNEL staining. **(F)** The expression of caspase-3, both full-length and cleaved (C-caspase-3) in colonic crypt cells, as assessed by western blot using antibodies against the respective proteins. The levels of Hsp90, a housekeeping protein, were measured as a loading control. Left: Immunoblot. Center and right: Graphs of data for protein expression, as obtained by measuring (using ImageJ) the band intensity in the western blot. The center graph shows values normalized to those for the Hsp90 loading control; the right graph shows ratio of cleaved:intact caspase-3. Data are presented as mean ± SEM; comparisons were conducted by two-tailed unpaired Student’s t-test. *p < 0.05, **p < 0.01, ***p < 0.001, n.s. no significant.

Dysfunction in the integrity of the intestinal barrier is known to contribute to gut inflammation^33^. Therefore, we investigated whether loss of Scribble promotes intestinal permeability, as assessed by fluorescein isothiocyanate (FITC) permeability. This experiment showed that, in DSS-induced *Scrib*^ΔIEC^ animals, the surface of the colonic mucosa mice accumulated higher levels of FITC-dextran (4000 Da), the serum exhibited higher levels of fluorescence, than did DSS-induced control mice (Fig. 3A). These results indicated that *Scribble* ablation in the IECs was associated with colonic leakage. Tight junctions (TJs) between IECs play a crucial role in maintaining the intestinal epithelial barrier function, and loss of TJ proteins increases the permeability of the intestine ^7^, permitting a variety of pathogenic bacteria to infiltrate gut tissues, leading in turn to severe inflammation. Therefore, we next examined the expression of known components of TJs. We found that the levels of key TJ proteins E-cadherin and occludin-1 were significantly decreased in the gastrointestinal tissues of *Scrib*^ΔIEC^ mice compared to control animals (Fig. 3B-C). At the same time, transmission electron microscopy (TME) revealed that *Scrib*^ΔIEC^ mice exhibited shorter tight junctions and wider gaps between epithelial cells (Fig. 3D). IECs are critical to maintaining the intestinal barrier function, and epithelial cell death is the most direct cause of barrier damage. We used terminal deoxynucleotidyl transferase dUTP nick end labeling (TUNEL) assays (a method for detecting DNA fragmentation associated with cell death) to demonstrate that the colons of DSS-induced *Scrib*^ΔIEC^ mice contained larger numbers of apoptotic cells than did those of DSS-expsosed *Scrib*^f/f^ mice (Fig. 3E). We also showed that cleaved caspase-3 protein (a markere of apoptosis) accumulated to significantly higher levels in the gastrointesinal tissues of DSS-induced *Scrib*^ΔIEC^ mice compared to those of DSS-exposed control animals (Fig. 3F). Together, these data suggested that the deletion of *Scribble* leads to barrier disruption and colonic leakage.

### Loss of *Scribble* in IECs disrupts the function of crypt stem cells in DSS-induced colitis

**Figure 4:**
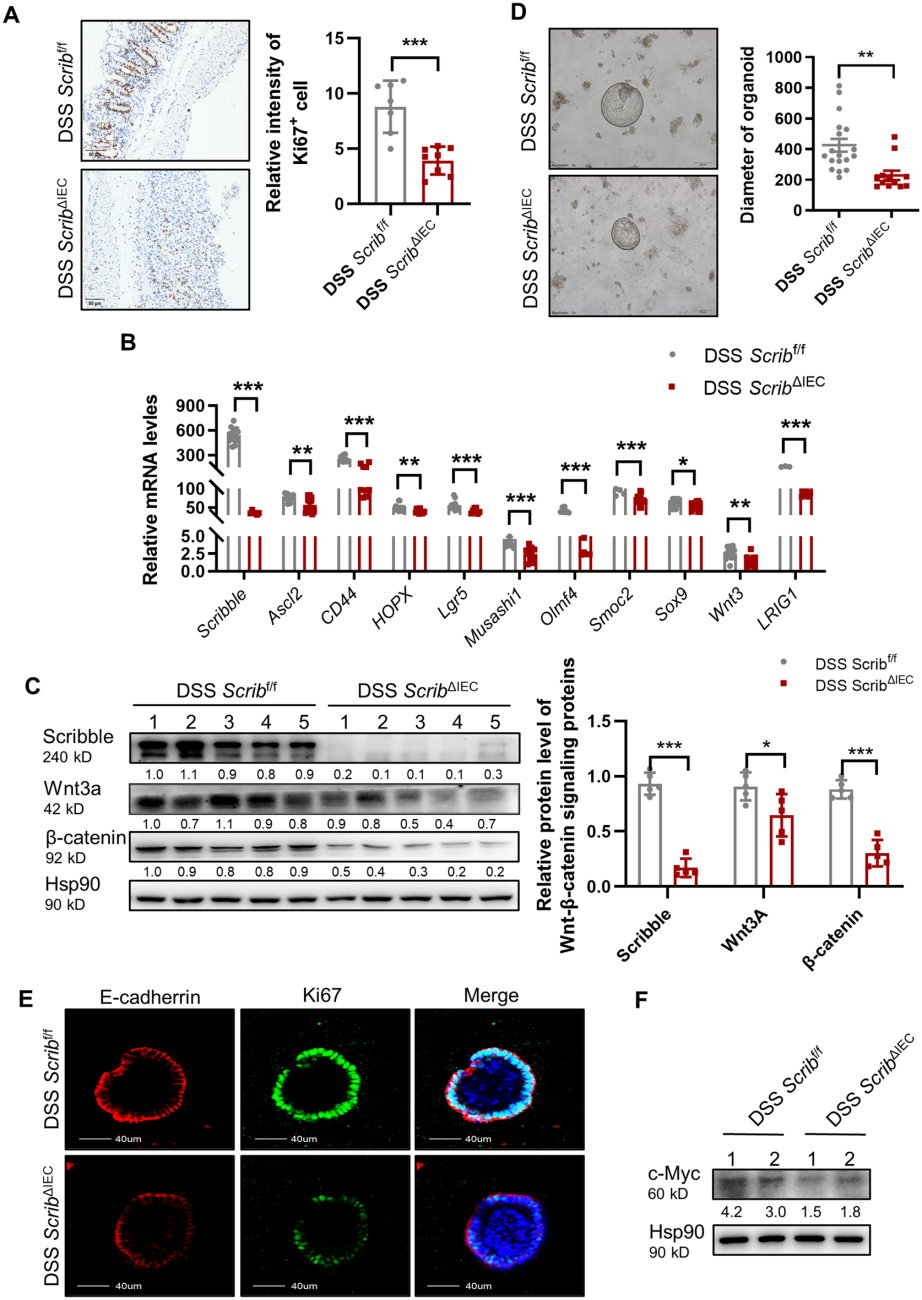
Loss of *Scribble* in IECs disrupts the function of crypt stem cells in DSS-induced colitis. Mice were provided with 7 days of free access to drinking water containing 2.5% DSS and then were euthanized for tissue collection. (**A**) IHC was performed to assess the expression of Ki67 in colon cells; the relative intensity of Ki67^+^ cells was measured using ImageJ. Left: Representative images of staining. Right: Graph of the relative intensity of staining for Ki67. (**B**) The levels of selected mRNAs (encoding markers of proliferation and stemness) in colon crypt cells were determined by qRT-PCR. (**C**) The expression of Wnt signaling-related proteins in colonic crypt cells were assessed by western blotting. Left: Representative blot strips following hybridization with antibodies against the indicated protein. Hsp90 was used as a loading control. Right: Plot of relative protiein levels as determined by western blotting; values were normalized to those of the loading control in the respective sample. (**D**) Left: Representative images of colon organoids derived from colonic crypts that had been isolated (on Day 7) from DSS-treated mice and subsequently cultured in vitro for 6 days. Right: The diameters of the resulting organoids were measured using ImageJ. (**E**) Representative images of IF staining for E-cadherin and Ki67 in colonic organoids derived from mice of the indicated genotypes. Left: staining for E-cadherin; center, staining for Ki67 (green); right, merged images. (**F**) The expression of c-Myc in colonic organoids was detected by western blotting. Representative blot strips are shown for membranes hybridized with antibodies against c-Myc and Hsp90 (loading control). Values between the strips correspond to relative c-Myc expression following normalization to values for the loading control in the respective sample. For all plots, data are presented as mean ± SEM; comparisons were conducted by two-tailed unpaired Student’s t-test. *p < 0.05, **p < 0.01, ***p < 0.001.

The gut is a rapidly renewing tissue;IECs are complete replaced every 5–7 days^34^. Therefore, a stable cycle of intestinal apoptosis and regeneration is essential for self-renewal and repair of the colon, and disruption of this process promotes the development of colitis. This balance between apoptosis and regeneration depends on crypt stem cells, and has a significant impact on the replacement of epithelial cells and maintenance of intestinal barrier integrity. Immunohistochemical (IHC) staining for Ki67, a biomarker of proliferation, revealed that the proportion of Ki67-positive cells was significantly reduced in *Scrib*^ΔIEC^ mice compared to control animals (Fig. 4A). Consistent with that observation, the expression in colon crypt cells of genes involved in proliferation and stem cell identity was significantly downregulated in *Scrib*^ΔIEC^ mice (compared to controls), including as *Lgr5*, *Ascl2*, *Smoc2*, and others (Fig. 4B). Wnt/β-catenin signaling also is known to be critical for the self-renewal of ISCs and mucosal repair. Using western blot analysis, we demonstrated that the levels of Wnt3a and β-catenin proteins were significantly decreased in colonic crypt cells of DSS-treated *Scrib*^ΔIEC^ mice compared to those from DSS-induced *Scrib*^f/f^ mice (Fig. 4C).

We extended this analysis by using colonic organoids, which are three-dimensional (3D) mini-guts derived from colonic stem cells or pluripotent stem cells^35^. Such organoids serve as a powerful model for the study of the self-renewal and proliferative abilities of ISCs. Specifically, we constructed colonic organoids derived from *Scrib*^ΔIEC^ and *Scrib*^f/f^ mice that had been treated with DSS. The ability of cells derived from *Scrib*^ΔIEC^ mice to form organoids was significantly impaired compared to that of cells derived from *Scrib*^f/f^ mice (Fig. 4D). We also performed immunofluorescent (IF) staining of these organoids, demonstrating that the expression of both Ki67 and E-cadherin was significantly decreased in the colonic organoid derived from *Scrib*^ΔIEC^ mice compared to those derived from *Scrib*^f/f^ mice (Fig. 4E). Next, we extracted proteins from the organoids and found that the levels of c-Myc, a key regulator of events downstream of the Wnt signaling pathway, were significantly decreased in *Scrib*^ΔIEC^ organoids compared to *Scrib*^f/f^ organoids (Fig. 4F). Together, these data suggested that *Scribble* deletion in IECs attenuates crypt stem cell proliferation in DSS-induced colitis.

### Loss of *Scribble* in IECs permits the accumulation of ROS as a result of decreased autophagy signaling

**Figure 5:**
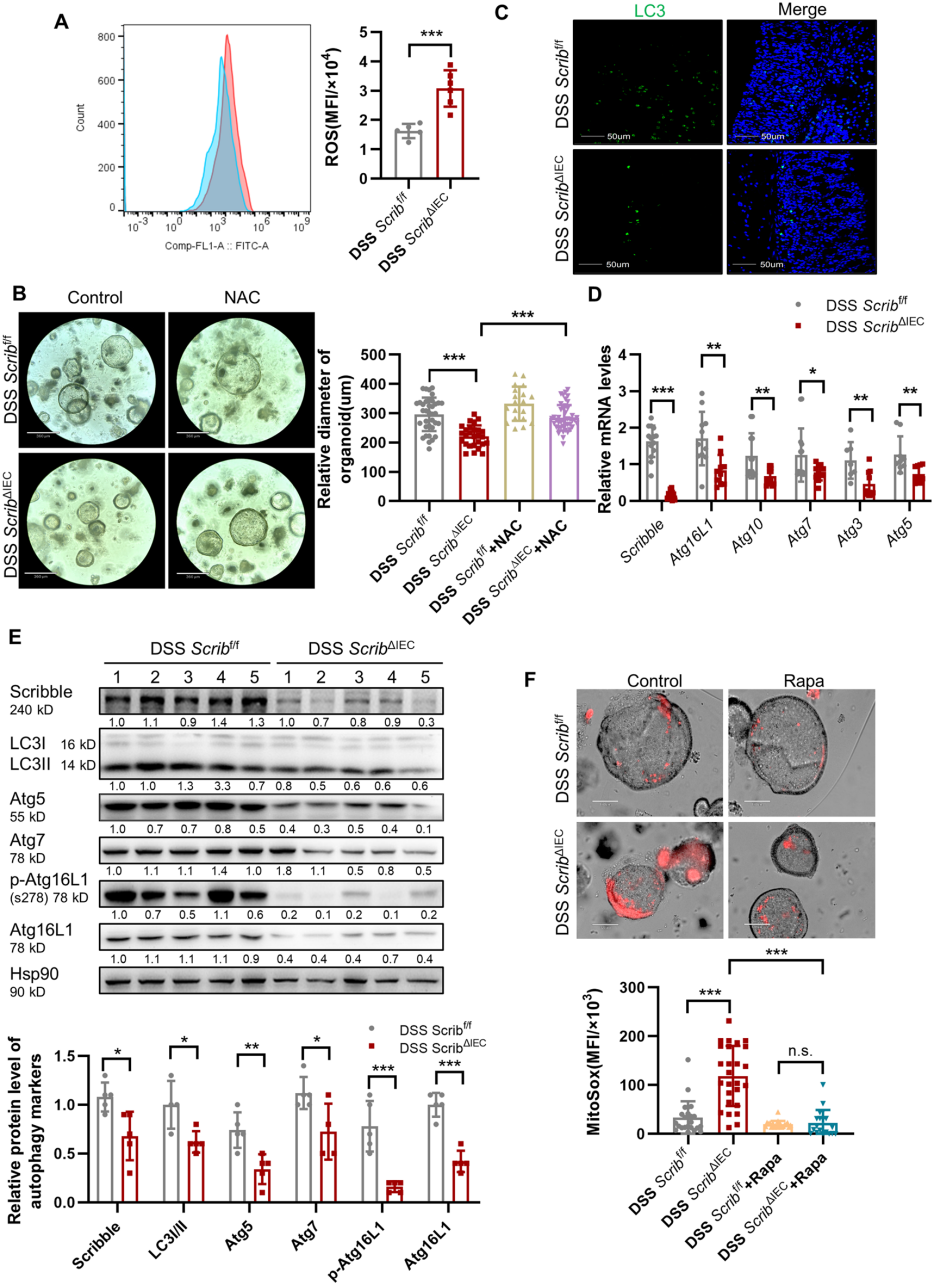
Loss of *Scribble* in IECs is associated with the accumulation of ROS and depletion of components of the autophagy signaling pathway. Colitis was induced by providing mice with 7 days of free access to drinking water containing 2.5% DSS; animals then were euthanized, colons were recovered, and colonic crypt cells were isolated. (**A**) Histograms (left) and MFI quantification (right) of ROS. (**B**) Left: Representative images of colonic organoids derived from tissues of DSS-treated mice were cultured for 5 days in the absence and presence of the antioxidant N-acetyl-l-cysteine (NAC). Right: The diameter of the resulting organoids were measured using ImageJ. (**C**) Representative images of vertical sections of colon samples follwing immunofluorescence (IF) staining for LC3 (green; left column) alone or merged with staining for DNA (DAPI; right column). (**D**) Relative mRNA levels of *Scribble* and autophagy-related genes in colonic crypt cells, as assessed by qRT-PCR. Values were normalized to those of a housekeeping gene. (**E**) The levels of Scribble and autophagy-related proteins was assessed by western blotting. Left: Representative blot strips following hybridization with antibodies against the respective proteins. Hsp90 was used as a loading control. Right: Relative expression as determined using ImageJ. Values were normalized to thatof the loading control (Hsp90) in the respective sample. (**F**) Colonic organoids were incubated (for 10 h on Day 3) in the absence (Control) or presence (Rapa) of 100 μM rapamycin. The organoids then were exposed to the MitoSOX dye probe. Left: Representative images of red fluorescence, photographed using an inverted microscope. Right: The fluorescence intensities of the organoids were measured and analyzed using ImageJ. For all plots, data are presented as mean ± SEM; comparisons were conducted by two-tailed unpaired Student’s t-test. *p < 0.05, **p < 0.01, ***p < 0.001, n.s. no significant.

Elevated ROS levels are known to decrease the number and quality of hematopoietic stem cells and muscle stem cells; oxidative stress plays an important role in apoptosis induction under both physiologic and pathologic conditions^36^. Therefore, we examined whether ROS levels in crypt cells are altered by the selective deletion of *Scribble*. For this assay, we incubated crypt cells from mice with DSS-induced colitis with 2′,7′-dichlorofluorescein diacetate (DCFH-DA), a dye that serves as a cell-permeable redox probe. Following entry into the cells, the parent chemical is oxidized to the fluorescent compound DCF upon exposure to ROS. As we hypothesized, fluorescence in DCFH-DA-exposed crypt cells derived from DSS-induced *Scrib*^ΔIEC^ mice was significantly higher than that in the DSS-induced control mice (Fig. 5A), indicating that *Scrib*^ΔIEC^ cells have elevated ROS levels. To determine whether impaired intestinal stem cell properties are related to the accumulation of ROS, we incubated organoid cultures with *N*-acetyl-l-cysteine (NAC, 100 µM), a known ROS scavenger. NAC promoted the growth of intestinal organoids (Fig. 5B), indicating that the accumulation of ROS inhibits the self-renewal of ISCs. Collectively, these data showed that ROS play a key role in Scribble-mediated colitis.

Next, we explored the mechanism by which *Scribble* knockout leads to ROS accumulation. Specifically , we investigated whether autophagy, a form of programmed death, is capable of limiting ROS accumulation by eliminating damaged or redundant mitochondria^37^. Autophagy is essential for maintaining intestinal homeostasis, proper intestinal immune response, and antimicrobial effects; abnormal autophagy can lead to the development of IBD^38^. We speculated that Scribble may influence the development of IBD by regulating autophagy. Consistent with this hypothesis, the expression of LC3, a marker of the autophagy, in gastrointestinal tissues was significantly decreased in *Scrib*^ΔIEC^ mice compared to *Scrib*^f/f^ mice (Fig. 5C). Moreover, the expression (at both the RNA and protein level) of autophagy-related genes in the crypt cells of *Scrib*^ΔIEC^ mice was significantly attenuated compared to that in *Scrib*^f/f^ mice, including the genes encoding LC3, Atg16L1, Atg5, Atg7, and (strikingly) p-Atg16L1, a recently proposed marker of autophagy (Fig. 5D-E). A similar effect was observed for the conversion of LC3B-I to LC3B-II (Fig. 5E), a process known to be essential to autophagy^39^ .

As a further test of the potential effect of Scribble on autophagy, we isolated IECs from the colons and subjected these cells to autophagy-inducing conditions consisting of culturing for 6 days following by incubation for 24 h in the presence of 100 ng.mL lipopolysaccharide (LPS). Western blotting analysis of the resulting cells revealed that the induced *Scrib*^ΔIEC^ IECs exhibit significantly decreases in the LC3II/LC3I ratio and in Atg16L1 compared to similarly treated *Scrib*^f/f^ cells (Supplementary Fig. 4A,B). Moreover, when SW480 cells (a human colon adenocarcinoma line) were engineered to overexpress of Scribble and then exposed to rapamycin (Rapa; 100 μM for 4 h, a condition known to induce autophagy^40^), the LC3II/LC3I ratio was elevated (compared to that in rapamycin-exposed control SW480 cells) (Supplementary Fig. 4C). Together, these results implied that knockout of *Scribble* inhibits autophagy.

To further verify whether the accumulation of ROS in *Scrib*^ΔIEC^ cells reflects defects in autophagy, we induced autophagy in colonic organoids by incubation (at Day 5 of growth) with Rapa for 10 h. MitoSOX, a red mitochondrial fluorescence probe, then was used to assess the level of ROS in organoids in which autophagy had been induced. Notably, in the absence of Rapa exposure, organoids derived from *Scrib*^ΔIEC^ mice accumulated significantly higher levels of ROS than did those derived from *Scrib*^f/f^ mice. However, when the organoids were treated with Rapa, the ROS signal in *Scrib*^ΔIEC^-derived organoids was significantly reduced (compared to no Rapa exposure), such that the difference between *Scrib*^ΔIEC^- and *Scrib*^f/f^-derived organoids was no longer significant (Fig. 5F). Together, theses results demonstrated that *Scribble* knockout causes dysregulation of autophagy, leading to the accumulation of ROS and (in turn) affecting the function of crypt cells.

### Scribble binds to Atg16L1 in an LRR domain-dependent manner, thereby inhibiting Atg16L1 degradation

**Figure 6:**
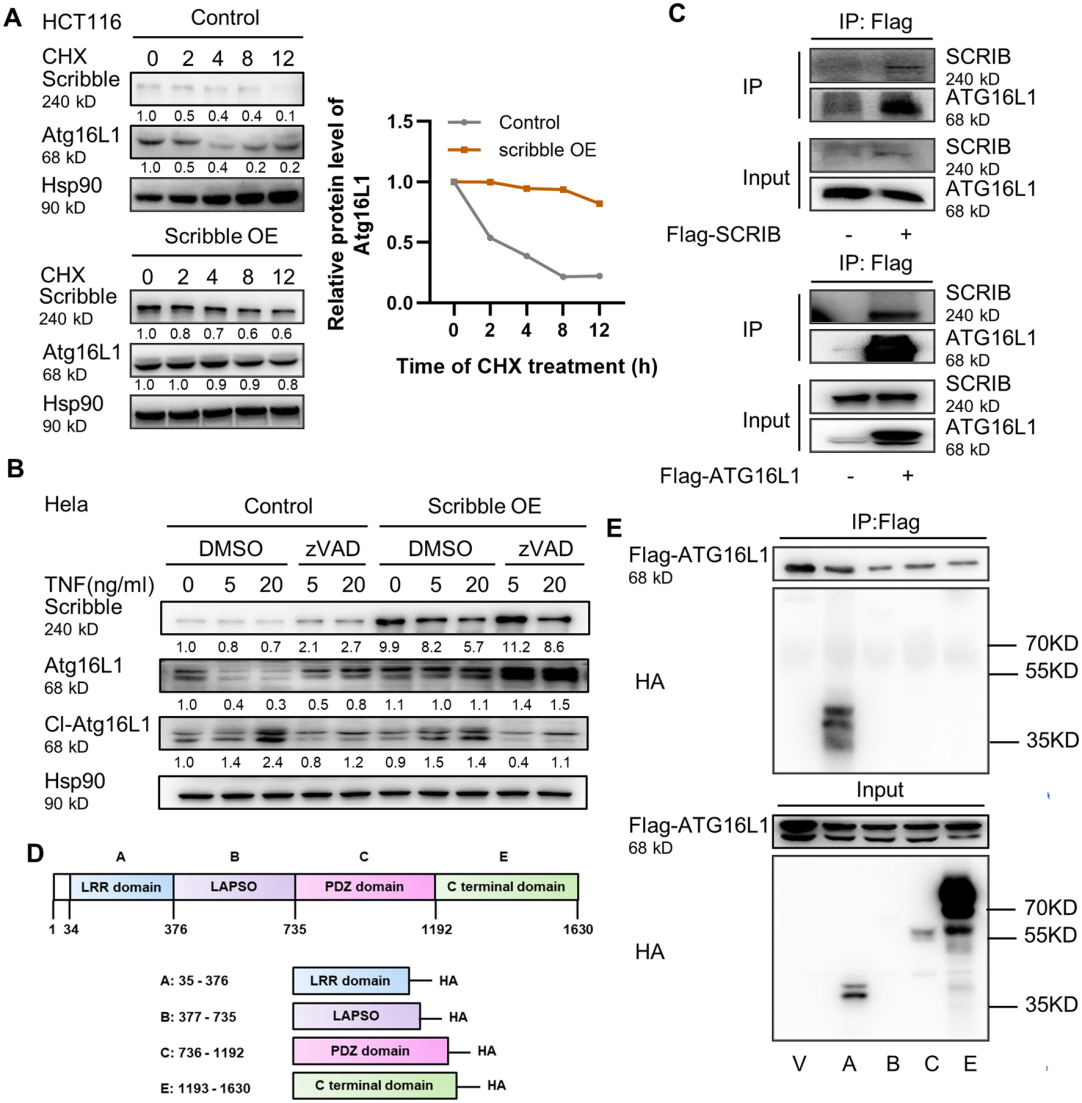
Scribble binds Atg16L1 in a LRR domain-dependent manner, thereby stabilizing Atg16L1 degradation. (**A**) Left: HCT116 cells were transfected with vector (Control) and Scribble-encoding plasmids, then incubated with the protein synthesis inhibitor cycloheximide (CHX, 100 μg/mL) for the indicated times; cells then were lysed, and the levels of Scribble and Atg16L1 were assessed by western blot. Representative images of blot strips are provided. Hsp90 was used as a loading control. Right: Relative intensities of band staining were measured and analyzed using ImageJ. Values were normalized to those of the loading control in the respective sample. (**B**) HeLa cells were transfected with vector (Control) or Scribble-encoding plasmids. Transfected cells were subjected to 1-h pretreatment with vehicle (DMSO) or 10 µM pan-caspase inhibitor (zVADfmk), then stimulated for 3 h with TNF in the presence of 10 µg/mL of the protein synthesis inhibitor cycloheximide (CHX). The cells then were lysed and the levels of Scribble, full-length Atg16L1, and proteolytically cleaved protein (Cl-Atg16L1) were assessed by western blot. Representative blot strips are shown; Hsp90 was used as a loading control. (**C**) Flag-tagged SCRIB (Flag-SCRIB) or Flag-tagged ATG16L1 (Flag-ATG16L1) was expressed in 293 T cells. Cells then were lysed and subjected to immunoprecipitation (IP) using anti-Flag-tag antibody. Proteins present before (Input) and after IP were visualized by western blotting with antibodies against the indicated proteins; representative images of blot strips are shown. − and + indicate the absence and presence (respectively) of the indicated Flag-tagged protein. (**D**) Schematic showing the full-length Scribble protein, and the four separate hemagglutinin (HA) -tagged Scribble subdomains (A: LRR, B: LAPSD, C: PDZ, D: C-terminal domain). (**E**) 293 cells already expressing Flag-tagged ATG16L1 (Flag-ATG16L1) were transfected with vector control (V) or plasmids encoding the indicated HA-tagged SCRIB protein domains. Cells then were lysed and subjected to immunoprecipitation (IP) using anti-Flag-tag antibody. Proteins present before (Input) and after IP were visualized by western blotting with anti-Flag-tag antibodies (to detect ATG16L1; smaller rectangular strips) or with anti-HA antibodies (larger square blots). Representative images of blots and strips are shown. The positions of size markers (in kDa) are shown to the right.

The above results demonstrated that Atg16L1 expression is significantly decreased in *Scrib*^ΔIEC^ mice. That observation is consistent with previous work showing the functional and clinical importance of ATG16L1 (the human homolog) in controlling intestinal epithelial homeostasis and inflammatory immune responses^41,42^. Therefore, we hypothesized that Scribble may influence autophagy by directly regulating Atg16L1. To further assess whether Scribble regulates Atg16L1 accumulation, a human colon cancer cell line (HCT116) engineered to overexpress Scribble was exposed to cycloheximide (CHX), an inhibitor of translation. We found that Atg16L1 protein had a longer half-life in Scribble-overexpressing cells than in control cells (Fig. 6A), which suggested that Scribble stabilizes the ATG16L1 protein. Protein degradation is mediated by pathways include ubiquitination-, proteasome- and caspase-dependent pathways. To identify the mechanism whereby Scribble affects the stability of Atg16L1, we first knocked down *Scribble* in HCT116 using a gene-specific small interfering RNA (siRNA), and then treated cells for 4 h with MG132, a known inhibitor of the proteasomal pathway. Notably, *Scribble* knockdown did not affect the proteasomal degradation of ATG16L1 (data not shown). Other work has shown that a specific mutation of the *Atg16L1* gene (the Atg16l1^T300^^A^ risk variant) renders the protein highly susceptible to proteolytic degradation by caspase 3^16,17^. To address the possible role of caspase-mediated degradation in the Scribble-dependent stabilization of Atg16L1, HCT116 was engineered to harbor a Scribble overexpression plasmid (or the empty vector control), pretreated for 1 h with 10 µM zVADfmk (a caspase inhibitor) or the vehicle control (dimethyl sulfoxide; DMSO), and then stimulated with varous concentrations of tumor necrosis factor (TNF) in the presence of CHX. In this model, Scribble overexpression inhibited the induction of Atg16L1 degradation by TNF; notably, the difference was eliminated by the addition of the caspase inhibitor. This result demonstrated that Scribble inhibits degradation of Atg16L1 by the caspase-dependent pathway (Fig. 6B).

Although Scribble is not known to possess any intrinsic enzymatic activity, the protein contains many critical domains that facilitate binding to other proteins. Co-immunoprecipitation (co-IP) of lysates from 293 T cells engineered to overexpress FLAG-tagged Scribble or Atg16L1 revealed that Scribble physically interacts with Atg16L1 (Fig. 6C). Subsequent in vitro pull-down assays using different hemagglutinin (HA) tagged-subdomains of the Scribble protein (designated here as A, LRR domain; B, LAPSD domain, C, PDZ domains; or D, C-terminal domain) indicated that Atg16L1 binds to the LRR domain of Scribble (Fig. 6D and E). These observations suggested that Scribble binds to Atg16L1 in a LRR domain-dependent manner to inhibit the caspase-mediated degradation of Atg16L1.

### Dietary sphingomyelin (SM) alleviates DSS-induced colitis and increases the expression of Scribble and autophagy-related genes

**Figure 7:**
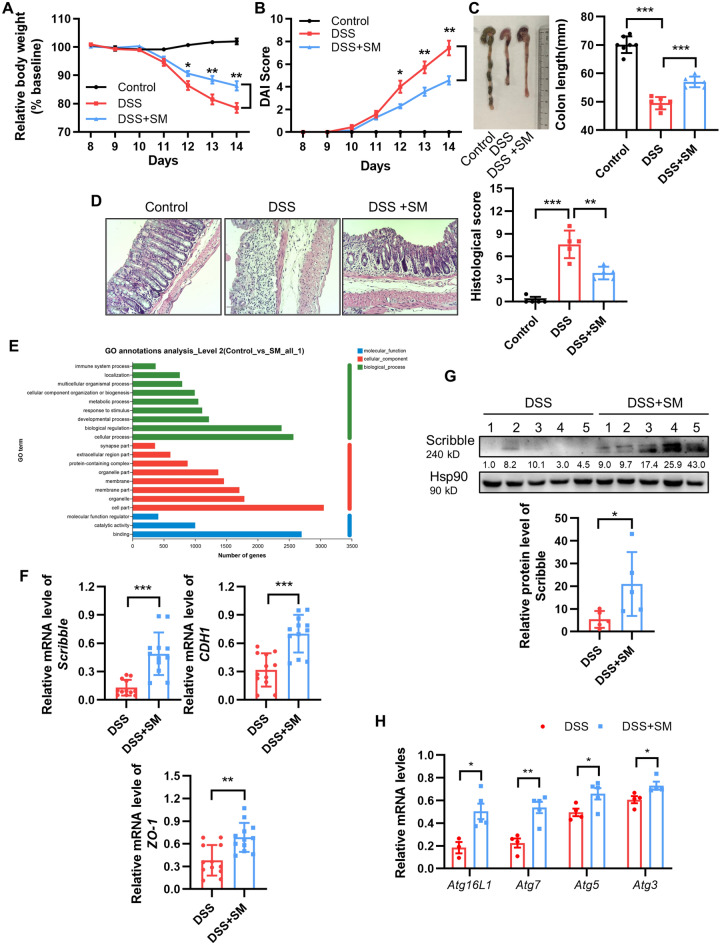
Dietary sphingomyelin (SM) alleviates DSS-induced colitis and is associated with increased expression (RNA and protein) of *Scribble* and autophagy-related genes. Colitis was induced in C57BL/6 mice by maintenance on DSS-containing water without or with sphingomyelin (SM) dietary intervention, and animals were euthanized on Day 14. Control mice were maintained on drinking water neat without dietary intervention. (**A**) Relative body weights (normalized to baseline). (**B**) DAI scores. (**C**) Left: Representative images of colons from experimental animals. Right: Graph of colon length data in the three groups. (**D**) Left: Representative images of H&E-stained colon tissue. Right: Graph of histological scores. (**E**) RNA was extracted from colon tissue and subjected to RNA-seq analysis. The graph shows the results of Gene Ontology (GO) annotation analysis from the comparison of DSS mice vs. DSS + SM animals. (**F**) RNA expression of *Scribble* and of the tight junction related genes *CDH1* and *ZO-1* was assessed by qRT-PCR. Values were normalized against the expression of a housekeeping gene. (**G**) Protein isolated from the colon tissue of DSS and DSS + SM mice was assessed for Scribble levels by western blotting. Left: Representative blot strips following hybridization with anti-Scribble antibody. Hsp90 was used as a loading control. Right: Blot band intensities were quantified and analyzed using ImageJ. Values were normalized to those of the loading control in the respective sample. (**H**) RNA was extracted from colon tissue and subjected to qRT-PCR analysis to assess transcript levels of the autophagy-related genes *Atg16L*, *Atg7*, *Atg5*, and *Atg3* were detected by qRT-PCR. Values were normalized to those of a housekeeping transcript in the respective samples. For all plots, data are presented as mean ± SEM; comparisons were conducted by two-tailed unpaired Student’s t-test. *p < 0.05, **p < 0.01, ***p < 0.001.

Food intake is known to be an important factor in the development of IBD. Studies have shown that intake of fruits and vegetables can decrease the risk of IBD^43^. We sought to explore what nutrients may have a mitigating effect on IBD. In a preliminary screen, we noted that sphingomyelin (SM) may ameliorate IBD (data not shown). SM, an animal membrane sphingolipid, is found primarily in milk, eggs, and soybeans^44^. SM and its metabolites exhibit a variety of biological activities, including the regulation of cell growth, differentiation, senescence, and apoptosis^45^. Recent studies have shown that dietary SM suppresses aberrant colonic crypt foci and lipid absorption^46,47^. To validate sphingomyelin’s possible role in counteracting DSS-induced colitis, mice were maintained on either of two diets: a control diet (AIN-93) or the same diet modified to contain 0.1% (wt/wt) milk SM (MSM; Avanti Polar Lipids). Following 1 week on either of these diets, mice were provided with 7 days of free access to drinking water neat or containing 2.5% DSS (Supplementary Fig. 5). Mice of the DSS + SM group displayed attenuation of weight loss, decreased DAI scores, longer colon lengths, and more-complete colon structure (as assessed by histopathology) than did with control (no SM) DSS mice (Fig. 7A-D) These results indicated that dietary SM ameliorates DSS-induced colitis.

To explore the mechanism of SM’s alleviation of colitis symptoms, we performed RNA-seq analysis on colonic tissue samples. Gene Ontology (GO) term analysis of the expression profiles indicated that the levels of transcripts encoding colonic inflammatory signals and cell junction proteins were significantly altered in DSS + SM animals compared to the control (no SM) DSS mice (Fig. 7E). Specifically, compared to control DSS animals, DSS + SM mice exhibited significantly decreased levels of the transcripts encoding pro-inflammatory cytokines including those for IL-1β, IL-6, and IFN-γ (Supplementary Fig. 6A,B). In contrast, the levels of transcripts encoding components of TJs, including the key genes *CDH1*, *ZO-1*, and *Scribble*, were significantly elevated in the DSS + SM mice compared to the control DSS animals; notably, the expression of *Scribble* was increased approximately fivefold in SM-fed mice (Fig. 7F). Western blot analysis confirmed the effects on Scribble expression, showing that also showed that the protein accumulated to significantly higher levels in DSS + SM mice (compared to control DSS animals) (Fig. 7G). Next, these experiments were extended to in vitro cultures (using HT29 and HCT116 cell lines) in which autophagy was induced (by exposure to 100 ng/mL LPS). For LPS-exposed cells, growth in the presence of SM resulted in dose-dependent manner increases in Scribble expression (compared to induced cells grown without SM supplementation) (Supplementary Fig. 6C,D). These studies indicated that SM alleviates DSS-induced inflammation in mice and increases the expression of TJ proteins, notably including Scribble. Perhaps more importantly, we observed that the increase in Scribble levels was accompanied by a significant increase in the expression of autophagy-related genes (Fig. 7H), further confirming the correlation between Scribble and autophagy.

## Discussion

**Figure 8:**
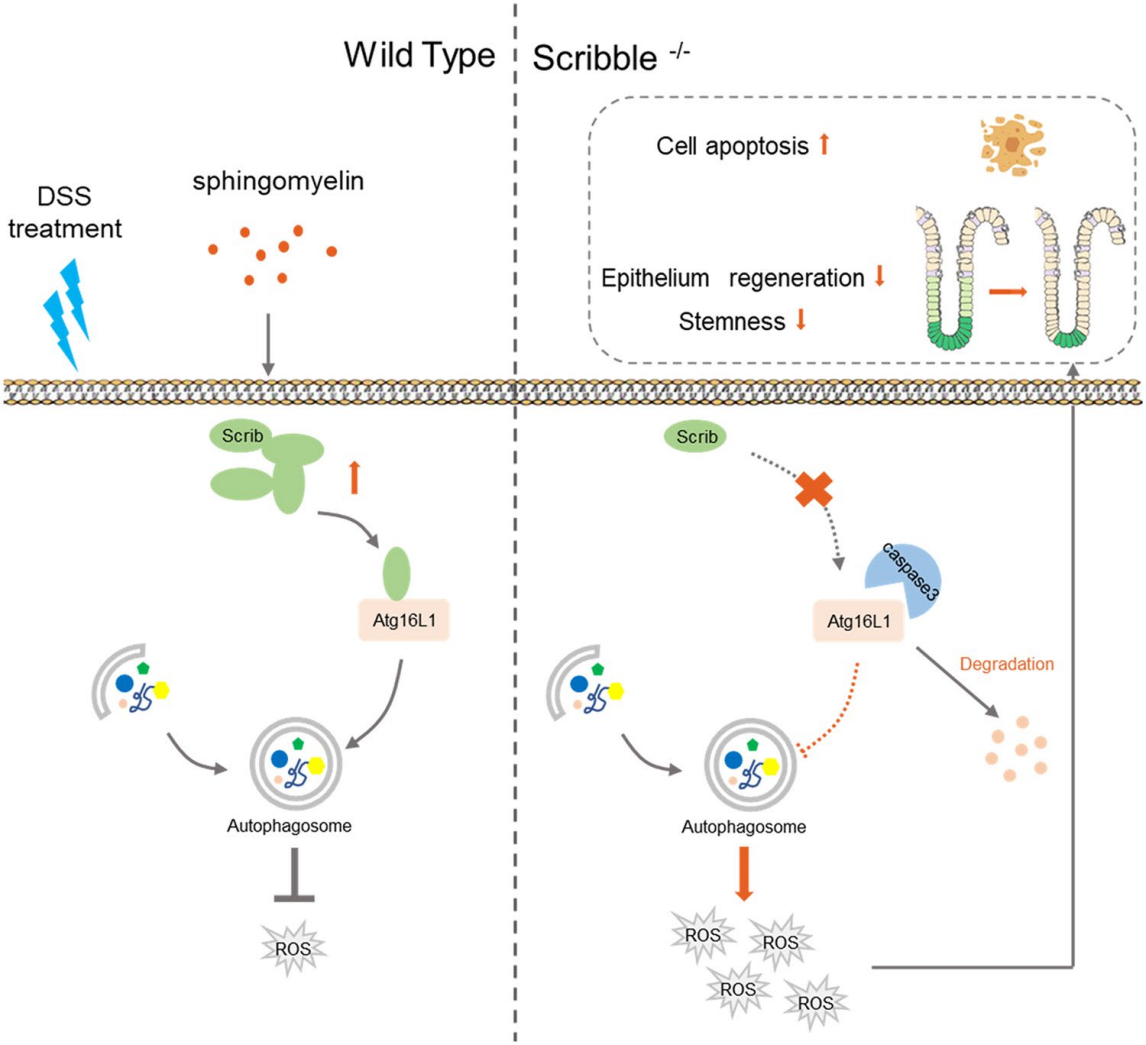
Proposed model for Scribble-mediated attenuation of ROS-induced damage to the colon. The polarity protein Scribble ensures normal levels of autophagy in intestinal cells by stabilizing the autophagy-related protein Atg16L1. Conversely, the deletion of *Scribble* in IECs leads to attenuation of autophagy, resulting in the accumulation of excess reactive oxygen species (ROS), and leading in turn to compromised intestinal barrier integrity and increased inflammation.

In the present study, we demonstrated that Scribble deficiency aggravates colitis by inhibiting autophagy, a process otherwise is used by the cell to respond to oxidative stress; the decrease in autophagy leads, in turn, to loss of integrity of the intestinal barrier, as shown schematically in Fig. 8. Our results indicate that Scribble may serve as a novel marker for maintenance of the intestinal barrier.

IBD seriously affects the quality of life of patients. The identification of effective targets for the screening, diagnosis, and treatment of IBD are expected to benefit the clinical treatment of patients with IBD. The intestinal epithelium is a single-cell layer that constitutes the most important "barrier" to the external environment; proteins currently used as biomarkers of intestinal barrier deficiency in IBD include Occludin, E-cadherin and so on. The intestinal epithelium is thought to maintain its integrity via a set of protein–protein complexes that contribute to the formation of a permeable barrier^34^. The polarity protein Scribble, an evolutionarily conserved protein belonging to the LAP (LRR and PDZ) family, plays a crucial role in the establishment of cell polarity and also is known to contribute to maintaining normal function of epithelia. In the present study, we evaluated the role of Scribble in the function of the intestinal barrier, using an in vivo model (DSS-induced colitis), in vitro cell culture, and intestinal organoids. Notably, these organoids have evolved as valuable tools for studying the regeneration function of the intestinal barrier. Our results indicated that Scribble is associated not only with the maintenance of intestinal integrity, but also with the regenerative functions of the colonic epithelium. We therefore sought to explore the possible mechanisms whereby Scribble contribute to intestinal epithelial homeostasis and to the repair of intestinal epithelial damage in IBD.

Autophagy is an evolutionarily well-conserved recycling process that is employed by cells in response to stress conditions, including ROS accumulation. Previous studies have shown that autophagy becomes dysregulated deleted for the genes encoding the autophagy proteins Atg16L1, beclin-1, or LC-3B. Other work has shown that normal levels autophagy are critical to the pathology of colitis. In the present work, we demonstrated that *Scribble* expression is decreased (at both the RNA and protein levels) in mouse model of DSS-induced colitis, suggesting Scribble plays a critical role in IBD. Further experiments revealed that selective deletion of *Scribble* in IECs leads to ROS accumulations, with subsequent IEC death and impaired ISC function, thereby disrupting the integrity of the intestinal barrier. Autophagy is considered the primary mechanism for removing ROS in cells^13^. We speculated that inhibition of autophagy may promote the accumulation of ROS in intestinal inflammation. Consistent with that hypothesis, our work revealed that *Scribble* deficiency resulted in depletion of Atg16L1, a key autophagy protein, reflecting decreased stability of the Atg16L1 protein. We further demonstrated that this decrease in stability resulted from increased caspase3-dependent degradation of the autophagy protein, a process normally impeded by the direct binding of Scribble to Atg16L1. Consistent with this inference, overexpression or knockdown of *Scribble* did not affect the ubiquitination of Atg16L1. A recent study in *Arabidopsis* also reported that 14-3-3, another polarity protein, contribute to autophagy by modulating degradation of an autophagy-related protein (ATG13), a process mediated by the SINAT E3 ubiquitin ligases^48^. Thus, our finding supports the idea that polarity proteins like Scribble may serve as molecular adaptors that help regulate autophagy by binding key components of the autophagy machinery (in this case, AtgL161) in tissues experiencing stress and inflammation. Thus, Scribble can be assigned a new function based on its ability to regulate the autophagy pathway.

We infer that Scribble contributes to IBD via Scribble’s regulatory role in autophagy. Furthermore, we demonstrated, using our in vivo model, that dietary supplementation with SM ameliorates the effects of DSS-induced colitis, and that this effect is mediated by increases in Scribble expression; these effects were confirmed using cell culture experiments. These observations suggest that targeting Scribble may provide a new treatment for colitis. However, our work also raises several issues. For instance, given the variety of sphingolipid structures, further work will be necessary to identify compounds that provide the greatest therapeutic effects. Additionally, the mechanism whereby SM potentiates Scribble expression needs to be elaborated. Our experiments also showed exposure to SM enhances the growth of organoids and facilitates the Wnt pathway (Supplementary Fig. 7A–C). It remains to be seen whether SM alleviates intestinal inflammation primarily via the Scribble-Atg16L1 axis or by other regulatory mechanisms. Finally, although we confirmed that Scribble stabilizes Atg16L, inhibiting caspase-dependent degradation of this autophagy protein, the mechanism of this inhibition is unknown. Other research has shown that IKKα protects Atg16L1 from caspase-dependent degradation by phosphorylating the autophagy protein at Ser278^49^. Further experiments will be needed to determine whether Scribble inhibits the Atg16L1 degradation by increasing IKKα-mediated phosphorylation of this target.

In conclusion, we provided the first (to our knowledge) demonstration that a polarity protein, Scribble, ameliorates IBD by autophagy-dependent sequestration of oxidative stress. Notably, the selective deletion of *Scribble* in IECs leads to abnormal autophagy, resulting in IEC death and decreased self-renewal and repair by ISCs, leading in turn to compromised integrity of the intestinal barrier and enhancement of colitis (Fig. 8). Furthermore, we demonstrate that SM, a key nutrient in eggs and milk, counteracts the development of colitis, an effect that may be mediated by increased expression of Scribble. We propose that Scribble may serve as a marker of the intestinal barrier status, an inference that may find application in the etiology, diagnosis, and treatment of IBD and related conditions.

## Methods

### Mice and mouse models

All mice were maintained in a specific pathogen-free (SPF) facility and all experimental procedures were approved by the Institutional Animal Care and Use Committee (IACUC) of the Shanghai Institute of Nutrition and Health (SINH) (Shanghai, China) and complied with the relevant guidelines for the care and use of experimental animals. To generate *Scrib*^ΔIEC^ mice, *Scribble*^flox/flox^ (*Scrib*^f/f^) mice were crossed with *Villin-cre* mice that express the Cre enzyme under control of the *Villin* promoter^50^.

For the dextran sulfate sodium (DSS) -induced colitis model, *Scrib*^ΔIEC^ and *Scrib*^f/f^ mice (8- to 12-week-old males) were provided with free access to drinking water containing 2.5% DSS (MW = 36–50 kD; MP Biomedicals, Santa Ana, CA, USA) for 7 days. Mouse weight and rectal bleeding were assessed daily. Colon length was measured following euthanasia. The disease activity index (DAI) and histological scoring were performed as described previously^51^.

### Histopathology, immunohistochemistry (IHC), immunofluorescence (IF), and TUNEL assay

To assess DSS-induced damage, formalin-fixed and paraffin-embedded murine colon tissue was sectioned (5-μm thicknesses) and stained with hematoxylin and eosin (H&E) stainby standard methods. For IHC staining, processing, sectioning, and staining were performed as described previously^52^; IHC employed antibodies directed against Ki67 (NBP1-40684, Novus). For IF staining, the primary antibodies were as follows: anti-LC3A/B (#12741, CST), anti-F4/80 (#70076, CST), anti-Ly-6G (#68590, CST), anti-E-cadherin (#14472, CST), and anti -c-Myc (#13987, CST). For the TUNEL assay, staining was performed using the TUNEL FITC Apoptosis Detection Kit (A111-01, Vazyme Biotech Co., Ltd., China) according to the manufacturer’s protocols.

### ROS staining by flow cytometry

For detecting ROS levels, crypt cells were incubated with CM-H2DCFDA (10 mM; C6827, Life Technologies), a cell-permeable detector of ROS. After incubation, the level of ROS was measured by flow cytometry (Beckman Coulter, CytoFlex LX). Gate strategy of ROS was used FITC; Histogram.

To quantify fluorescence, mean fluorescence intensity (MFI) of ROS staining was determined and analyzed using FlowJo software (v10.8.1). The mean value was used for subsequent statistical analysis.

### Colon organoid culture

Using sterile technique, 6- to 8-week-old *Scrib*^ΔIEC^ and *Scrib*^f/f^ mice were euthanized by intraperitoneal injection with 1.25% 2,2,2-tribromoethanol (0.2 mL/10 g of body weight). The colons then were recovered and any fecal material was removed by flushing with phosphate-buffered saline (PBS). The entire colon was turned over to make the crypt outward, then rinsed three times with cold PBS containing 2% (vol/vol) penicillin and streptomycin (Gibco™, 15140163, 10000U/ml). The entire colon was placed in a 5-mL tube filled with 4 mL of Cell Recovery Solution Buffer (Catalog No. 354253; Corning, NY, USA) and incubated for 30–40 min at 2–8 °C. Subsequently, the crypt structures were obtained by filtering the digested tissue through 70-μm strainers. The crypt structures were then washed (via 4 to 5 cycles of centrifugation (2 min at 200 × g at 2–8 °C) and resuspension) in 10 mL DMEM/F12 (Gibco™, Waltham, MA) supplemented with 1% (vol/vol) penicillin and streptomycin (Gibco™^)^, 15140163, 10000U/ml), 1% Glutamax (Gibco™), and 1% (vol/vol) 4–1-piperazineethanesulfonic acid (HEPES) buffer (1 M, Gibco™^)^), until remained clear after centrifugation. The crypts were counted and resuspended in Matrigel (354,230, BD) to a density of ~ 600 organoids/mL, then seeded in prewarmed 24-well culture plates at 500 μL/well. After incubation at 37 °C for 10 min, the Matrigel was completely solidified, and colon organoid complete medium (consisting of a 1:1 mixture of DMEM/F12:L-Wnt3A supernatant supplemented with 20% (vol/vol) fetal bovine serum (FBS; BI), 1% (vol/vol) penicillin and streptomycin, 500 ng/mL R-Spondin (Catalog No. 3474-RS, R&D), 50 ng/mL epidermal growth factor (EGF; Catalog No. 50482-M01H, Sino Biological Co., Ltd., China), 100 ng/mL Noggin (Catalog No. 250-38-20, Peprotech), and 10 μM Y-27632 (Catalog No. 1254, Tocris)) was added at 500 μL/well. The plates then were incubated at 37 °C for the indicated times, and the resulting organoids were collected for analysis by western blot or IF. For the N-acetyl-L-cysteine (NAC) treatment, NAC (Sigma-Aldrich) was added to the growth medium to a final concentration of 100 µM on Day 1. For rapamycin (Rapa; MCE (Med Chem Express), Rapa was added to the growth medium to a final concentration of 100 µM on Day 5. Six hours later, organoids were incubated with MitoSOX Red probe (5uM; Invitrogen). The L-Wnt3A cell line, which overexpresses Wnt3A, was purchased from Shanghai Fuxiang Biotechnology Co., Ltd.

### Isolation of intestinal epithelial cells (IECs)

Four-week-old *Scrib*^ΔIEC^ and *Scrib*^f/f^ mice were euthanized and the colons were recovered, cut into fragment, and washed 4-5 times with PBS (room temperature). The cleaned pieces then were resuspended in a digestion solution consisting of DMEM medium supplemented with 5% (vol/vol) FBS, 0.8m g/mL collagenase XI (Sigma), 4 μg/mL dispase (BD Biosciences, San Jose, CA), and 2% (vol/vol) penicillin and streptomycin and incubated for 3–4 h at 37 °C. The digested mixture was centrifuged for 5 min at 300 ×*g* and rinsed 5 times with cold wash buffer (DMEM medium supplemented with 2.5% (g/vol) D-sorbitol (Sigma), 2% (vol/vol) penicillin and streptomycin, and 20% (vol/vol) FBS). The resulting isolated IECs were seeded on Matrigel-coated 6-well plates and cultured in IEC growth medium (DMEM/F12 supplemented with 1% (vol/vol) insulin mix, 2.5% (vol/vol) FBS, and 2% (vol/vol) penicillin and streptomycin). For lipopolysaccharide (LPS; LPS from *E. coli* 0111: B4, L2630, Sigma-Aldrich) treatment, IECs were cultured for 4–5 days before the addition of LPS to a final concentration of 100 ng/mL; the plates then were incubated at at 37 °C for the indicated time.

### Colon epithelial permeability assay

*Scrib*^ΔIEC^ and *Scrib*^f/f^ mice that had been treated with DSS (to induce colitis) were administered by oral gavage (PO) with fluorescein isothiocyanate (FITC)-dextran (Sigma-Aldrich Cas#60842-46-8) at 0.6 mg/g body weight; 4 h later, animals were euthanized and colon tissue and serum were collected. Colon tissue was frozen and cryosectioned for use in the assay. Serum was subjected to various dilutions in PBS and distributed to a 96-well plate at 100 μL/well. The FITC content of the serum was determined by a fluorometer (excitation 490 nm, emission 520 nm) to measuring fluorescence in the dark.

### Immunoprecipitation (IP)

IP was performed as described previously^52^. Briefly, 293 T cells (ATCC, cultured using DMEM medium with 10% FBS in a 37 °C incubator) were transfected with the indicated plasmids and harvested 24–48 h later. Cells then were combined with IP Lysis Buffer (25 mM Tris–HCl (pH 7.4), 150 mM NaCl, 1% (vol/vol) NP-40, 1 mM EDTA, 5% (vol/vol) Glycerol) and incubated for 30 min at 4 °C. The resulting lysates were incubated with pull-down hemagglutinin (HA) -conjugated beads (or with control beads) for 1 h at 4 °C. Following incubation, the supernatant was discarded and the beads were washed 3 times with IP Wash Buffer (50 mM Tris (pH7.5), 274 mM NaCl, 1% (vol/vol) Triton X-100, 5 mM EDTA, 10% Glycerol, 10 mM NaF). The Wash Buffer then was discarded, and the beads were resuspended in 30 μL IP Lysis Buffer and 30 μL 2 × Loading Buffer and incubated for 10 min at 100 ℃ to release the proteins. The resulting samples were stored frozen at − 20 ℃ pending analysis by western blot.

### Western blot analysis

Western blot analysis was performed as described previously^52^. Before hybridization with the primary antibody, each of the protein bands were cut from the whole membranes according to the predicted size of actual proteins by prestained protein marker (Shanghai YaMei Biomedical Technology Co., LTD.), and a bright-field image and exposure image of each band were performed with the Tanon Western blot imager (Tanon 5200 Multi). For the current study, the primary antibodies were as follows: anti-p-NF-κB (Catalog No. 3033, CST), anti-HSP90 (Catalog No. 4875, CST), anti-p-Stat3 (Catalog No. 9145, CST); anti-Scribble (Catalog No. 3193, CST), anti-E-cadherin (Catalog No. 14472, CST), anti-occludin (Catalog No. 91131, CST), anti-LC3A/B (Catalog No. 12741, CST), anti-Atg16L1 (Catalog No. 8089, CST), anti-Atg5 (Catalog No. 12994, CST), anti-Atg7 (Catalog No. 8558, CST), and anti-p-JNK (Catalog No. 4668, CST). Quantitative analysis of protein levels was obtained by gray-scale analysis of band intensity using ImageJ software (version 4.1; National Institutes of Health and the Laboratory for Optical and Computational Instrumentation (LOCI, University of Wisconsin))^53^. Relative expression levels were determined by normalizing protein levels to that of the loading control in the respective sample.

### Real time-polymerase chain reaction (PCR) and quantitative reverse transcription-PCR (qRT-PCR)

Total RNA from cells or tissues was isolated using the Trizol reagent (Takara, Dalian, China) according to the manufacturer’s protocols. RNA glue (gel imaging) was used to detect its integrity of the extracted RNA, and measured the concentration of the extracted RNA using a NanoDrop2000 ultra microspectrophotometer and ensured that the OD260/OD280 ratio (R) was in the range of 1.8–2.1, which indicate minimal protein contamination of the RNA. 1 μg RNA was reverse transcribed by using the PrimeScript RT-PCR Kit (Takara Bio) according to the manufacturer’s instructions. The resulting complementary cDNA then was subjected to real-time PCR using SYBR Premix Ex Taq (Takara Bio) according to the manufacturer’s instructions. Gene-specific primer sequences are provided in the Supplementary Table.

### Statistical analysis

Statistical analyses were performed using ImageJ and Prism (v. 6.0; GraphPad) software. Comparisons of data from two groups were conducted using the two-tailed unpaired Student's t-test. *P* < 0.05 was considered statistically significant.

### Supplementary Information

Supplementary Figures.

Supplementary Tables.

## Data Availability

All data relevant to this study are included in the main article or uploaded as supplementary information. Source data may be obtained from the corresponding author on reasonable request.
