# Supplementary material for: Scribble deficiency mediates colon inflammation by inhibiting autophagy-dependent oxidative stress elimination
Source: Sci Rep. 2023 Oct 26;13:18327. doi: 10.1038/s41598-023-45176-2 (PMC10603050; doi:10.1038/s41598-023-45176-2)
Supplement: Supplementary file 1 — Supplementary Figures. [file 41598_2023_45176_MOESM1_ESM.docx]

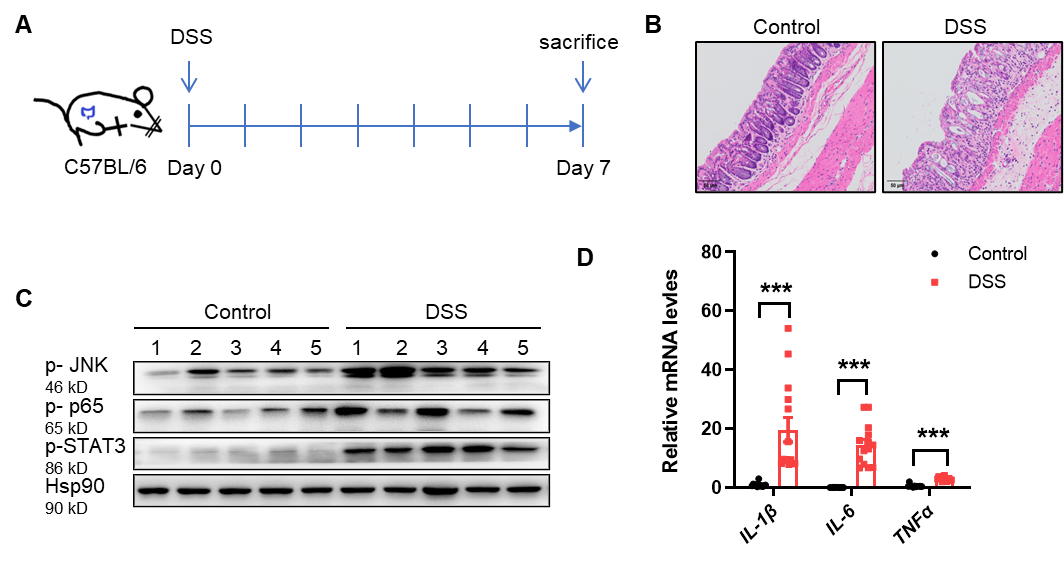


**Supplementary Figure 1. The model of DSS -induced colitis.** The total samples of two groups were 8 (Control) and 8 (DSS) respectively. **(A)**The model of DSS-induced colitis in C57BL/6 mice. C57BL/6 mice (male, 8-week-old) were divided into two group and provided with drinking water (Control) or drinking water with 3% DSS (DSS) for 7 days respectively. Day 0 is the initiation of DSS treatment. **(B)**The images of H&E staining in control group and colitis group. **(C)** Western Blot was performed to detect the expression of P-JNK、P-NF-κB、P-STAT3 in colon tissue. **(D)** qRT-PCR analysis of mRNA expression of inflammatory cytokines *IL1β*、*IL6*、*TNFα* in distal colonic tissues. The data are presented as mean ± SEM. Statistical analyses were conducted using two-tailed unpaired Student’s t-tests, * *p* <0.05, ** *p* <0.01, *** *p* <0.001.


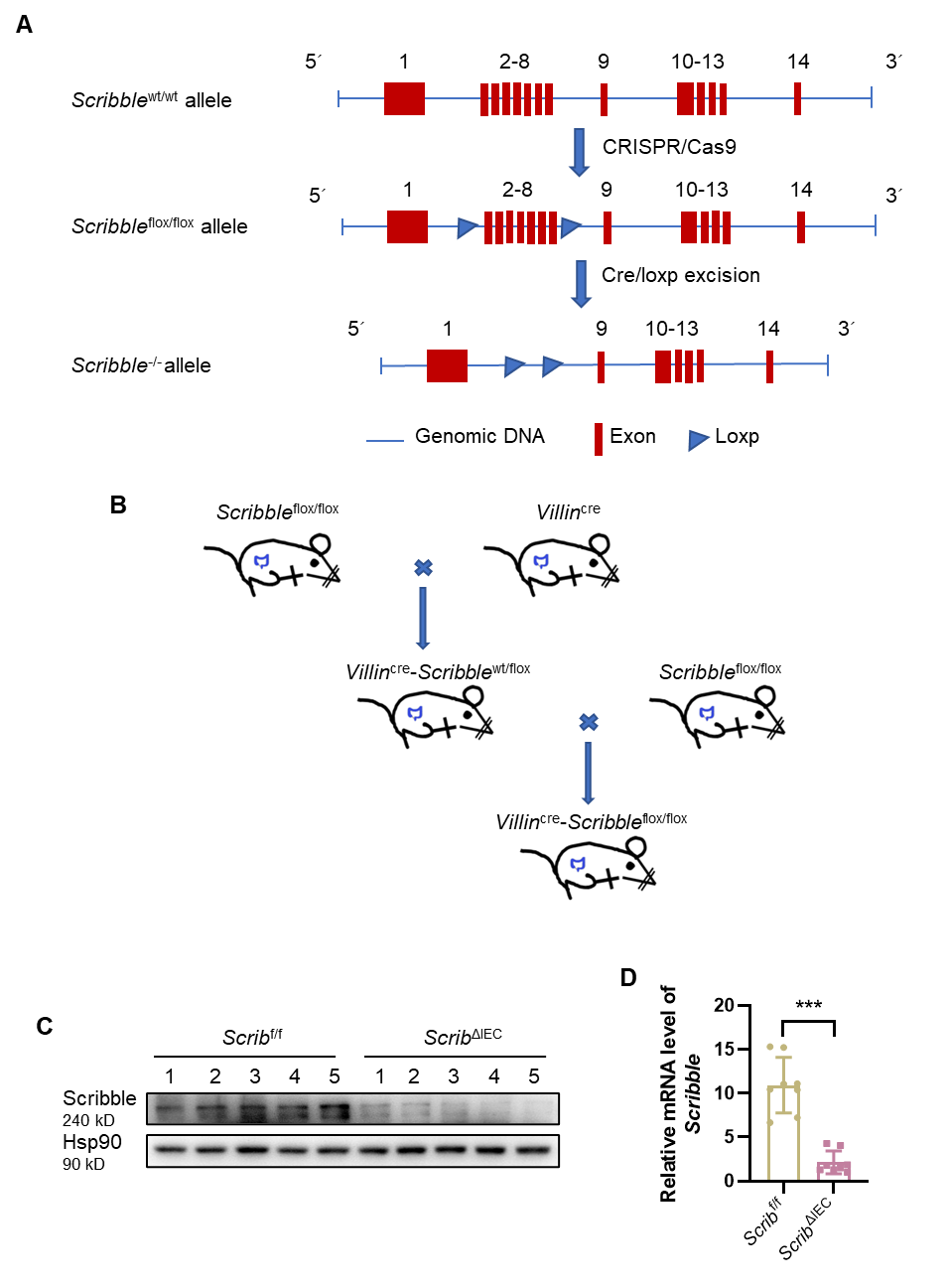


**Supplementary Figure 2. The construction and expression of Scribble in intestinal-specific-*Scribble* knockout mice. (A)**The schematic diagram of constructing *Scribble* knockout mice. **(B)** Hybridization of *Scribble* intestinal-specific knockout mice. **(C)** Western blot and **(D)** qRT-PCR analysis of Scribble in colonic tissues. The data are presented as mean ± SEM. Statistical analyses were conducted using two-tailed unpaired Student’s t-tests, * *p* <0.05, ** *p* <0.01, *** *p* <0.001.


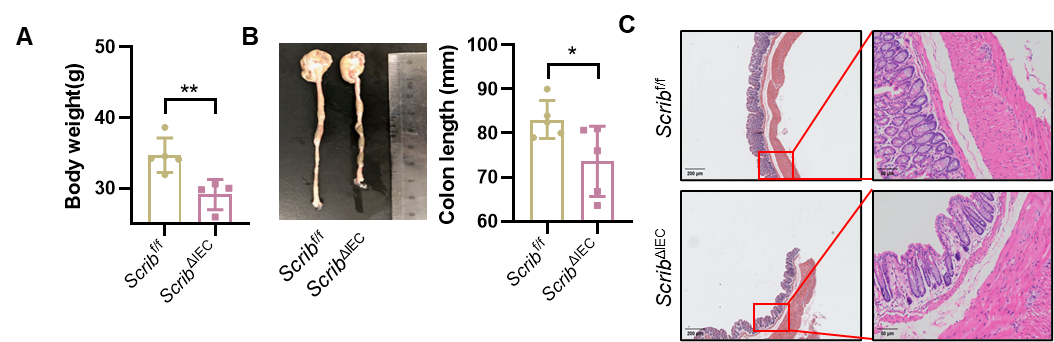


**Supplementary Figure 3. Loss of Scribble in IECs causes colon injury. (A)** Body weight, **(B)** colon length and **(C)** HE images of colon tissue from *Scrib*^f/f^ and *Scrib*^ΔIEC^ mice (8 months old). The data are presented as mean ± SEM. Statistical analyses were conducted using two-tailed unpaired Student’s t-tests, * *p* <0.05, ** *p* <0.01, *** *p* <0.001.


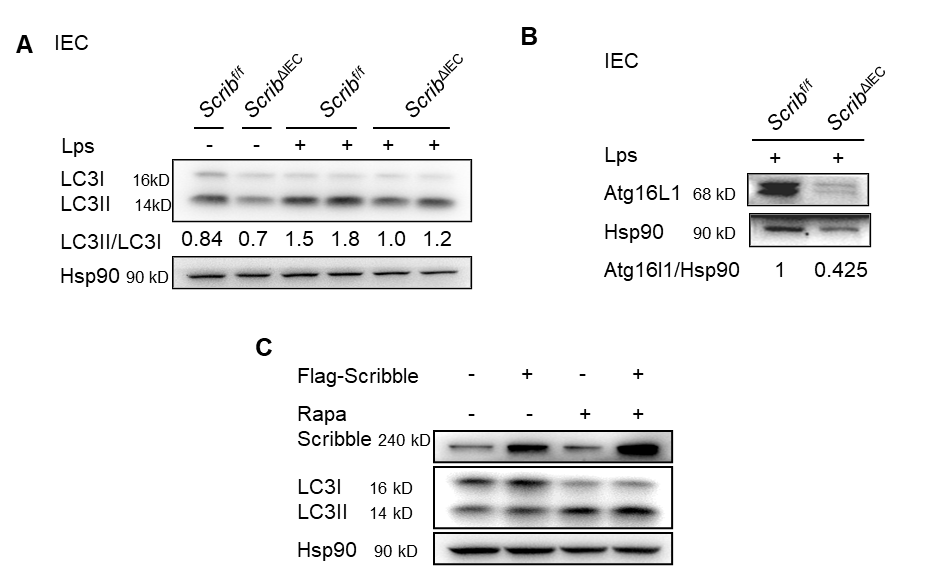


**Supplementary Figure 4. *Scribble*-knockout suppresses autophagy and the expression of Atg16L1.** IEC isolated from *Scrib*^f/f^ and *Scrib*^ΔIEC^ were extracted and cultured in vitro for 6 days, and then stimulated with 100ng/ml LPS for 24 h. **(A)** The expression of LC3 and Atg16L1**(B)** was determined by Western Blot. **(C)** SW480 cells transfected with control or Scribble plasmid were treated with Rapa (100uM) for 4h. LC3 was detected by immunoblotting.


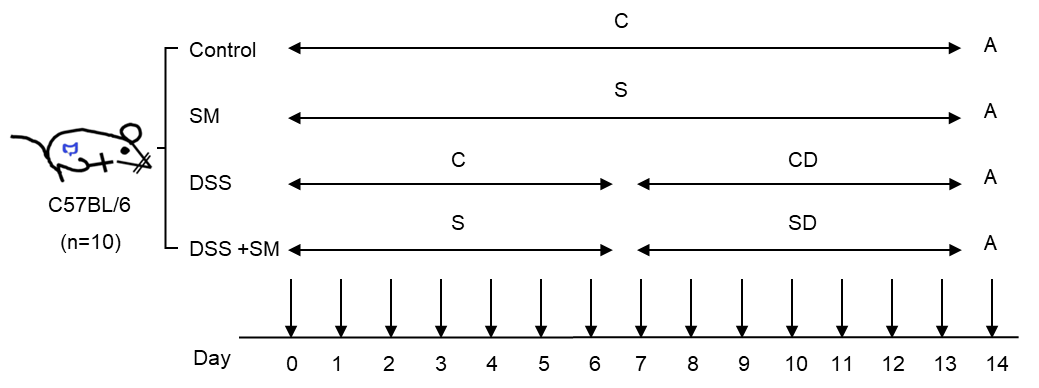


**Supplementary Figure 5. The model of DSS-induced colitis with sphingomyelin diet intervention in C57BL/6 mice.** C: control diet (AIN-93), S: AIN-93 with 0.1% sphingomyelin, D: 3% DSS, A: Autopsy.


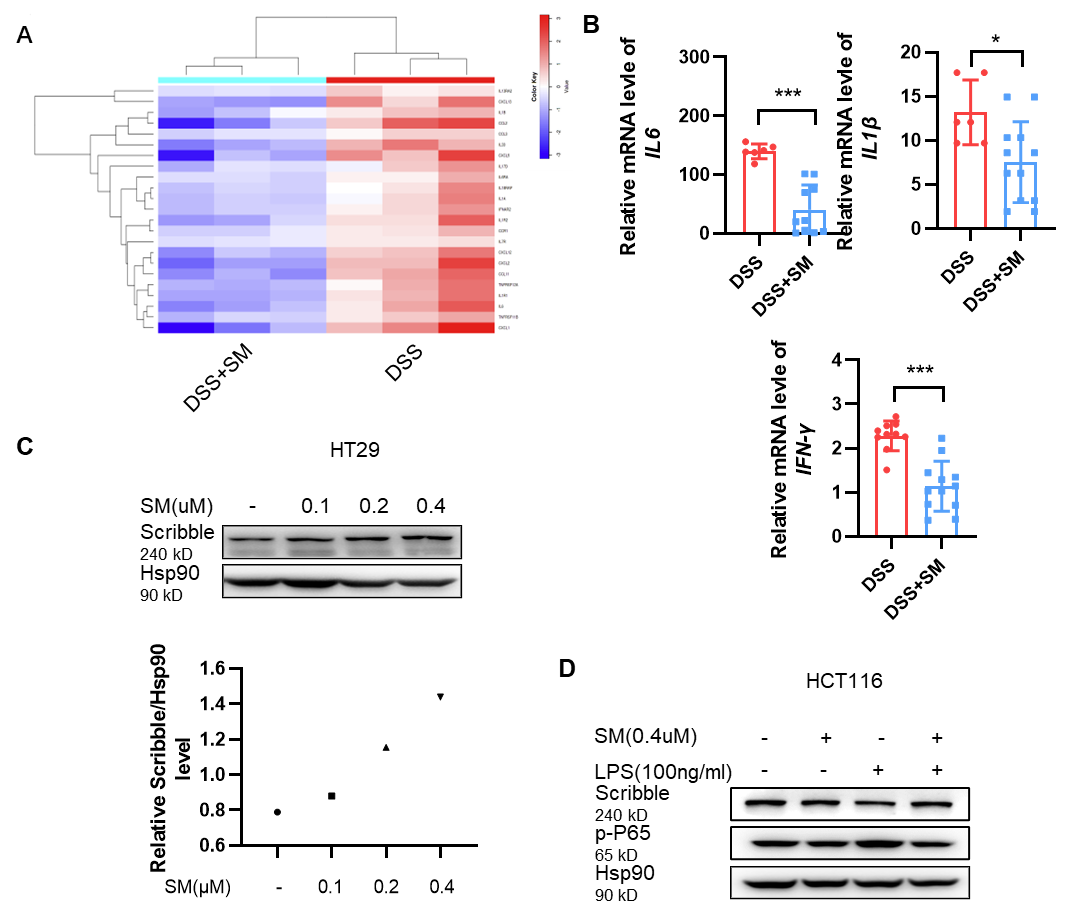


**Supplementary Figure 6. SM decreases intestinal inflammation and increases the expression of Scribble in DSS-induced colitis. (A)** Heatmap showing relative expression intensity of >4 fold differentially expressed cytokines in the colon between the DSS and the DSS+SM group. **(B)** The expression of pro-inflammatory cytokines *IL6*、*IL1β*、*IFN-γ* in transcription levels by qRT-PCR. (C) The expression of Scribble in HT29 cells, which were treated with different concentration of sphingomyelin (SM) in the presence of LPS (100ng/ml). Gray scale statistics were performed by Image J. (D) The expression of Scribble and p-NF-κB in HCT116 cells with or without SM and LPS. The data are presented as mean ± SEM. Statistical analyses were conducted using two-tailed unpaired Student’s t-tests, * *p* <0.05, ** *p* <0.01, *** *p* <0.001.


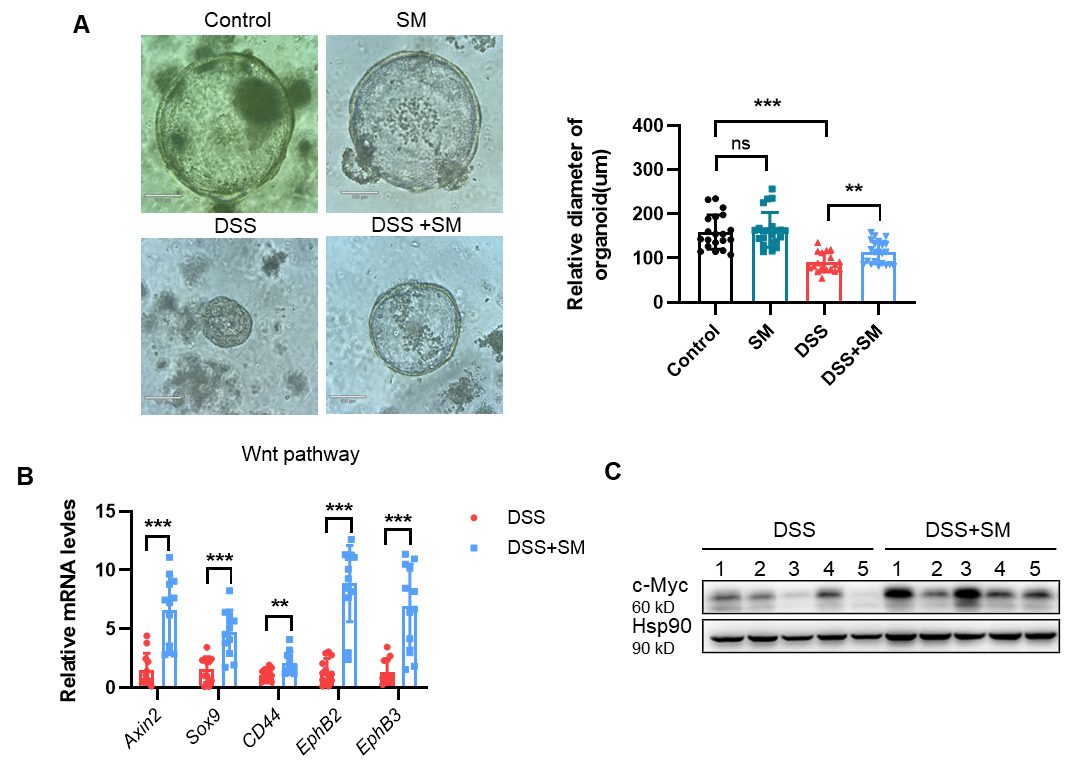


**Supplementary Figure 7. Dietary Sphingomyelin enhances the growth of organoid. (A)** Representative images of colon organoids derived from colonic crypt that were isolated from indicated-treated mice and then cultured in vitro for 4 days (right), and the diameter of organoids were measured by Image J. **(B)** The expression of Wnt pathway markers in isolated small colonic crypts qRT-PCR. **(C)** The expression of c-Myc in colonic crypt were detected by Western Blot. The data are presented as mean ± SEM. Statistical analyses were conducted using two-tailed unpaired Student’s t-tests, * *p* <0.05, ** *p* <0.01, *** *p* <0.001.
